# Diagnostic performance of deep learning for leg length measurements on radiographs in leg length discrepancy: A systematic review

**DOI:** 10.1002/jeo2.70080

**Published:** 2024-11-10

**Authors:** Bradley A. Lezak, James A. Pruneski, Jacob F. Oeding, Kyle N. Kunze, Riley J. Williams, Michael J. Alaia, Andrew D. Pearle, Joshua S. Dines, Kristian Samuelsson, Ayoosh Pareek

**Affiliations:** ^1^ NYU Langone Orthopedics NYU Langone Health New York New York USA; ^2^ Department of Orthopaedic Surgery Tripler Army Medical Center Honolulu Hawaii USA; ^3^ Mayo Clinic Alix School of Medicine Rochester Minnesota USA; ^4^ Sports Medicine and Shoulder Service, Department of Orthopedic Surgery Hospital for Special Surgery New York New York USA; ^5^ Department of Orthopaedics, Institute of Clinical Sciences, The Sahlgrenska Academy University of Gothenburg Gothenburg Sweden

**Keywords:** artificial intelligence, deep learning, diagnostic imaging, machine learning, orthopaedics

## Abstract

**Purpose:**

To systematically review the literature regarding machine learning in leg length discrepancy (LLD) and to provide insight into the most relevant manuscripts on this topic in order to highlight the importance and future clinical implications of machine learning in the diagnosis and treatment of LLD.

**Methods:**

A systematic electronic search was conducted using PubMed, OVID/Medline and Cochrane libraries in accordance with Preferred Reporting Items for Systematic Review and Meta‐Analysis guidelines. Two observers independently screened the abstracts and titles of potential articles.

**Results:**

A total of six studies were identified in the search. All measurements were calculated using standardized anterior‐posterior long‐leg radiographs. Five (83.3%) of the studies used measurements of the femoral length, tibial length and leg length to assess LLD, whereas one (16.6%) study used the iliac crest height difference to quantify LLD. The deep learning models showed excellent reliability in predicting all length measurements with intraclass correlation coefficients ranging from 0.98 to 1.0 and mean absolute error (MAE) values ranging from 0.11 to 0.45 cm. Three studies reported measurements of LLD, and the convolutional neural network model showed the lowest MAE of 0.13 cm in predicting LLD.

**Conclusions:**

Machine learning models are effective and efficient in determining LLD. Implementation of these models may reduce cost, improve efficiency and lead to better overall patient outcomes.

**Clinical Relevance:**

This review highlights the potential of deep learning (DL) algorithms for accurate and reliable measurement of lower limb length and leg length discrepancy (LLD) on long‐leg radiographs. The reported mean absolute error and intraclass correlation coefficient values indicate that the performance of the DL models was comparable to that of radiologists, suggesting that DL‐based assessments could potentially be used to automate the measurement of lower limb length and LLD in clinical practice.

**Level of Evidence:**

Level IV.

**Abbreviations**
AMA: anatomical mechanical angle
AP: anterior‐posterior
CNN: convolutional neural network
DL: deep learning
ICC: intraclass correlation coefficient
LAMA: leg angle measurement assistant
LLD: leg length discrepancy
LLR: long‐leg radiographs
MAD: mechanical axis deviation
MAE: mean absolute error
MFC: medial femoral condyle
mLDFA: mechanical lateral distal femoral angle
mLDTA: mechanical lateral distal tibia angle
mLPFA: mechanical lateral proximal femoral angle
mMPTA: mechanical medial proximal tibia angle

## INTRODUCTION

Anatomic leg length discrepancy (LLD) is a common condition in which the length of one leg is different from the other. This condition can be congenital or acquired [6], and estimated prevalence values of LLD as high as 90% have been reported, with an average magnitude of 5.2 mm [12]. This can cause problems with gait, balance and overall physical function, and is often addressed with either shoe lifts or surgical procedures such as epiphysiodesis, limb lengthening (distraction osteogenesis), internal magnetic lengthening rod) and/or amputation and prosthetic fitting [1, 3, 38, 40]. Regardless of the cause, LLD can significantly impact an individual's quality of life and ability to perform everyday activities. Machine learning has the potential to significantly improve the diagnosis and treatment of LLD in orthopaedic surgery [2, 17, 41]. One way that machine learning can be utilized in this context is through the development of systems for automatically analyzing and reporting on leg length radiographs, as described by Larson et al. [17] These systems can quickly and accurately assess limb length, allowing for more efficient diagnosis and treatment planning. This can be especially beneficial in cases where LLD is not immediately apparent, as it can be time‐intensive for healthcare professionals to manually measure leg length using traditional methods for all patients. In addition to automating the measurement process, machine learning can be used to measure LLD based on bilateral iliac crest height difference, as outlined by Kim et al. [11] This method of measurement can be particularly useful in cases where other methods are not feasible, such as when an individual has severe scoliosis or other abnormalities of the spine [28].

Deep learning (DL), a type of machine learning, can also be used to assess knee alignment in lower extremity radiographs as a means of diagnosing LLD, as demonstrated by Simon et al. [36] Differing heights of the knees can often be an indication of a difference in leg length and using DL to assess knee alignment can help healthcare professionals identify LLD more accurately and efficiently [2]. In addition to these applications, machine learning can be used to streamline the measurement process and reduce the risk of human error through the use of computer‐aided systems for automatically measuring leg length on full‐leg radiographs, as described in the study by Lee et al. [18] These systems can accurately and consistently measure leg length, ensuring that patients receive the most accurate and appropriate treatment. Another way that machine learning can be utilized in the context of LLD is through the use of convolutional neural networks (CNNs), a type of machine learning algorithm, to locate anatomical landmarks on radiographs for the purpose of measuring LLD, as outlined in the paper by Tsai et al. [39] This can be especially useful in cases where traditional methods of measurement are difficult or impossible to use, such as when an individual has severe deformities or abnormalities. Finally, DL can also be used to measure LLD in children based on radiographs, as demonstrated in the study by Sze et al. [41] By identifying and addressing LLD early on, healthcare professionals can help ensure that children have the best possible outcomes in terms of physical function and quality of life.

The use of machine learning is established in orthopaedics [4, 5, 10, 11, 13, 17, 18, 19, 22, 23, 24, 25, 26, 27, 30, 31, 32, 33, 36, 37, 39, 41] and has the potential to greatly improve the diagnosis and treatment of LLD leading to improved outcomes for patients. Specific pathologies where this may be relevant include but are not limited to, congenital limb length discrepancies due to hemihypertrophy, proximal femoral deficiency, developmental dysplasia of the hip, congenital talipes equinovarus, as well as paralytic disorders, infection, trauma and tumour. The purpose of this systematic review was to investigate and assess the literature regarding machine learning in LLD and to provide insight into the most prominent manuscripts on this topic in order to highlight the importance and future clinical implications of machine learning in the diagnosis and treatment of LLD.

## METHODS

### Identification and selection of articles

A systematic electronic search in accordance with the 2009 Preferred Reporting Items for Systematic Review and Meta‐Analysis statement [29] was conducted using PubMed, OVID/Medline and Cochrane libraries. The time frame for the search was the conception of each online database until 1 January 2024. The following Boolean search syntax was used to conduct the search: ‘leg length discrepancy’ AND ‘deep‐learning’ OR ‘machine learning’. Studies populated from the search met inclusion criteria if (a) the study methods and analyses were relevant to the use of DL for detecting and/or quantifying LLD and (b) was published in the English language. Studies were excluded if data was only published in the form of a conference abstract, editorial, technique paper, cadaveric or animal study, or letter to the editor. We did not exclude any studies on the basis of the level of evidence.

Two observers (B. L. and A. P.) independently screened the abstracts and titles of potential articles. The full‐text review was only performed during the study selection process if necessary to determine whether the articles satisfied inclusion and exclusion criteria. A total of 10 records were initially identified, and a total of six were ultimately included in the qualitative analysis as per inclusion criteria. There were no duplicates found.

### Data acquisition

All data were recorded into a custom spreadsheet using a modified information extraction table [8]. Categories for data collection for each full article included (1) article information; (2) input features; (3) data set size; (4) ground truth labels; (5) DL models used; (6) Output Measurements; (7) Output Classes; (8) Pretrained Models (if Used); (9) Size of training, validation and test sets; (10) Performance; (11) Other metrics (e.g., efficiency, failure rate).

### Assessment of heterogeneity and methodologic quality

Modified Methodological Index for Nonrandomized Studies scoring criteria were used to assess quality as has been previously applied in systematic reviews on artificial intelligence (AI) in orthopaedics [15, 16], Quality appraisal focused on the identification of (1) a clear study aim, (2) description of inclusion and exclusion criteria for input features (all eligible imaging examples included), (3) determination of ground truth (reference standards for AI), (4) report of distribution of data set (training, validation and testing phases), (5) described how performance of AI model was assessed (area under the curve [7]/prediction) and (6) clearly described AI model used. Each study could score a total of seven points, with a score of zero indicating poor methodologic quality, and a score of seven indicating the highest methodological quality. Two independent observers (B. L. and A. P.) assessed all included studies. The interobserver reliability was excellent. Any discrepancies were resolved by consensus.

### Statistical analysis

All data were qualitatively synthesized and reported in both narrative fashion in addition to table format. Extracted data were presented as means and ranges when appropriate with associated *p* values, given the degree of heterogeneity between studies. All studies considered a *p* < 0.05 to indicate statistical significance. All data extraction and analyses were performed in Microsoft Excel (Microsoft Corporation).

## RESULTS

A total of six studies were identified in the search (Table 1) [11, 17, 18, 36, 39, 41]. All six studies were included in the qualitative data analysis. All measurements were calculated using standardized anterior‐posterior (AP) long‐leg radiographs (LLRs). Five (83.3%) of the studies used measurements of the femoral length, tibial length and leg length to assess LLD [17, 18, 36, 39, 41], whereas one (16.6%) study used the iliac crest height difference to quantify LLD [11].

**Table 1:** Six studies on LLD using ML/DL were included in the qualitative analysis.

Study	Input features	Data set size	Ground/truth label assignment	AI models used	Output classes	Output measurements	Pretrained CNN	Training/validation set	Size training set	Size/validation set/validation method	Size test set	Performance	Other metrics
Larson et al. [17]	AP LLR	863	3 MSK trained radiologists	fasterRCNN‐RestNet101 for anatomic landmarks; EfcientNet‐D0 for hardware detection	Native, hardware	Leg length, femur length, tibia length, mechanical axis angle, pelvic tilt		643 annotations; 7591 anatomic landmarks			220	Absolute mean error: Femur and tibia leg lengths: <1%. Mechanical axis angle and pelvic tilt: <1°. Hardware detection accuracy: 99.8%. ICCs: Leg Length: 1.00, Femur length: 0.99, tibia length: 1.00, Mech axis: 0.98, Pelvic tilt: 0.86	
Kim et al. [11]	AP LLR	1598	One radiologist, one radiology resident	U‐Net 1″ Coarse segmentation of the upper pelvic bone. U‐Net 2: fine segmentation of the iliac crests		Iliac crest height difference			1038	260	300	Absolute Difference: Perfect (<1.3 mm): 187 (65%); Good (<10 mm): 93 (32%); Fair (10–20 mm): 4 (25%)	
Simon et al. [36]	AP LLR	295	Two orthopaedic surgeons		HKA angle, AMA, JCLA, mLDFA, mechanical‐lateral distal tibia angle mLDTA, mLPFA, mMPTA, MAD, leg length, femur length, tibia length	LAMA Software (Version 1.03.17, IB Lab, GmbH)			Mean absolute error: femoral and tibial lengths: 0.39 cm; leg length: 0.32 cm; LLD: 0.41 cm. Pearson correlation coefficient: femoral and tibial lengths: 0.99; leg length: 0.99; LLD: 0.92.			ICC: leg length: >0.99. Mean absolute deviation: HKA: 0.39 (0.35–0.44)°; mLDFA: 0.96 (0.76–1.18)°; mMPTA: 1.07 (0.95–1.22)°; leg length: 5.00 (4.50–5.70) mm; LLD: 0.90 (0.80–1.00) mm	LAMA failed readings: 6 (2%)
Lee et al. [18]	AP LLR	2767	One radiologist	DL‐Cascade Architecture: Stage 1: ROI detection with single‐shot multibox detector. Stage 2: 12 DL segmentation models (U‐Net) Stage 3: Conventional image‐processing algorithms. Code is accessible at https://hithub.com/RTOSChansu/llm/.	Femoral length, tibia length, leg length, LLD				1895	472	400		Machine reading time: 8.68 ± 0.18 sec
Tsai et al. [39]	AP LLR	504	One paediatric radiologist	CNN	Femoral length, tibia length, leg length, LLD					124; four‐fold cross‐validation		Mean absolute error: Femoral length: 0.33; tibia length: 0.30 cm; limb length: 0.30 cm and LLD: 0.41 cm	Average machine reading time: 0.26 sec
Zheng et al. [41]	AP LLR	179	One paediatric radiologist, one radiology fellow	CNN. Code accessible at https://github.com/zhengq-github/leg-length-discrepancy-study.git	Femoral length, tibia length, leg length, LLD				70	32	77	Mean Absolute Error: Femoral and tibial lengths: 0.45 cm; limb length: 0.45 cm and LLD: 0.51 cm	Average manual measurement time: 96 sec. Average machine measurement time: 1 sec

Abbreviations: AMA, anatomical‐mechanical angle; AP, anterior‐posterior; CNN, convolutional neural network; DL, deep learning; HKA, hip–knee–ankle; JCLA, joint‐line convergence angle; LAMA, leg angle measurement assistant; LLD, leg length discrepancy; LLR, long‐leg radiographs; MAD, mechanical axis deviation; ML, machine learning; mLDFA, mechanical‐lateral distal femoral angle; mLPFA, mechanical‐0‐lateral proximal femoral angle; mMPTA, mechanical medial proximal tibia angle; MSK, musculoskeletal.

### Model performance: Femoral, tibial and leg length

Five of the studies reported femoral length, tibial length and leg length as separate measurements with associated performance metrics [17, 18, 36, 39, 41]. A total of three studies (50%) reported mean absolute error (MAE) as a performance metric when comparing model performance to the ground truth labels. The MAE for these DL models ranged from 0.14 to 0.39 cm for femoral length, 0.11 to 0.4 cm for tibial length and 0.15 to 0.45 cm for leg length. The remaining two studies (40%) reported intraclass correlation coefficient (ICC) values to indicate agreement between radiologist measurements and machine predictions. These values are traditionally defined as 0.9 excellent; ≥0.75–0.89 good; ≥0.5–0.74 moderate and <0.49 poor reliability [14]. The two studies reported ICCs for measurements of length ranging from 0.98 to 1.0.

Larson et al. [17] used a Faster‐RCNN—ResNet 101 model from the open‐source TensorFlow Object detection library, which has been successfully used in the past for automatic detection of knee osteoarthritis [20]. The group trained their model on 643 LLRs that were annotated by students and reviewed by a fellowship‐trained radiologist. Their annotated points included the femoral head apex, femoral head centre, ischium, intercondylar notch, tibial spine and ankle mortise on each extremity. From these points, they were able to calculate leg length, femoral length and tibial length. On their test set of 220 images, the model predictions yielded ICCs ranging from 0.99 to 1.00 for all length measurements compared to the aggregate ground truth label.

Simon et al. [36] used the Leg Angle Measurement Assistant (LAMA) software (version 1.03.17, IB Lab GmbH) system for their analysis of 295 LLRs. LAMA is a commercially available model that annotates angle and length measurements on LLRs and is trained on more than 15,000 radiographs compiled from several single and multicentre studies. LAMA automatically localizes anatomic features of the femur, tibia and a calibration mark to assess the required landmarks to measure leg length, femur length and tibia length. On their test set, including 295 LLRs, the predicted measurements demonstrated excellent (ICC > 0.99) reliability when compared to the ground truth measurements annotated by two independent orthopaedic surgeons.

In their work, Lee et al. [18] describe a cascade DL architecture composed of three stages that they used to assess LLD. First, they used a single‐shot multibox detector [21] to detect 12 regions of interest (ROIs). After classifying the ROI, they used 12 separate U‐net models [35] to segment each ROI. Finally, using the bone contours derived from the segmentations, they were able to use an image‐processing algorithm to identify relevant landmarks in the pelvis, knee and ankle. They trained their DL system using 1895 LLRs, and ground truth labels were annotated by a single board‐certified radiologist. On their test set including 400 patients, they reported MAE values ranging from 0.13 to 0.15 cm for femur, tibia and leg length.

Tsai [39] developed a CNN using a total of 504 LLRs. The author used the method proposed by He et al. [9] for model fitting, and stochastic gradient descent with momentum was used to optimize the neural network [34]. The author, a board‐certified paediatric radiologist, served as the ground truth annotator for comparison. Uniquely, the author used k‐fold cross‐validation to evaluate model performance. With *k* = 4, the LLRs were broken into four groups, with one group being the testing data set. This process was repeated three times, with each group serving once as the test set and yielded total MAEs ranging from 0.30 to 0.33 cm when aggregated.

Zheng et al. [41] also used a CNN in their work. First, for coarse segmentation, the radiographs were split into left and right‐leg images. Subsequently, with fine segmentation, the femurs and tibias were segmented on the now unilateral full‐limb images. After the fine segmentation, leg length was measured by calculating the distance between the extracted corresponding pixels on the edges of the segmentation masks. For comparison, manual segmentation masks were annotated by a radiology postdoctoral fellow and a paediatric radiologist. This model was trained on 70 LLRs, fine‐tuned on 32 images and ultimately tested on 77 radiographs. They reported an MAE of 0.45 for femur length, tibial length and full‐leg length on their testing data.

### Model performance: LLD

In addition to quantifying individual bone length and full‐leg length, three (50%) of the studies reported measurements of LLD directly, with associated performance metrics [11, 39, 41]. Tsai [39] used the CNN mentioned in the previous section to predict the location of the femoral head, medial femoral condyle (MFC) and tibial plafond. The model output was a prediction heatmap for these anatomical landmarks, and the mean point error across all patients in the study for the location of the top of the femoral head, MFC and centre of the tibial plafond were 0.37, 0.39 and 0.42 cm, respectively. Using these points, the author was able to predict individual leg lengths and quantify the LLD in a subsequent step. With this method, Tsai was able to predict the LLD with an MAE of 0.41 cm across all four folds. Notably, the ICC for LLD compared to ground truth labels across all folds was 0.88, compared to ICC values > 0.98 for femoral, tibial and limb length.

Similarly, Zheng et al. [41] used their CNN to automate LLD calculations by calculating the distance between pixels at the edge of their segmentations. They defined the femoral length as between the tangents to the apex of the femoral head and the convexity of the MFC. The tibial length was between the convexity of the MFC to the tibial plafond. The leg length was defined as the distance from the top of the femoral head to the tibial plafond. These distances were compared between extremities to output a prediction for estimated LLD. With this approach, their MAE for LLD on their test set was 0.51 cm. Of note, the Pearson correlation for LLD compared to the ground truth labels was 0.92, compared to 0.99 for femoral, tibial and leg length.

Kim et al. [11] developed two U‐Net models to automatically measure the iliac crest height difference on LLRs. The first U‐Net was trained for coarse segmentation of the upper pelvic bone, and the second U‐Net was trained for the fine segmentation of the iliac crests. The final model outputs segmentations of the iliac crest, which are overlaid on the LLRs. Subsequently, the iliac crest height difference was automatically calculated as the difference between the highest y‐coordinates from each iliac crest. For comparison, two independent ground truth reviewers (one paediatric radiologist and one radiology resident) measured the distance from the imaginary horizontal line parallel to the ground to the bilateral iliac crest's highest points and subtracted the left iliac crest height from the right to quantify the LLD manually. This model was trained on 1038 LLRs, validated/fine‐tuned on 260 radiographs, and tested on 300 LLRs. The authors reported absolute differences of (<1.3 mm): in 187 (65%) images, <10 mm in 93 (32%) images and 10–20 mm in four (25%) images. The authors reported 16 failed readings, most commonly attributed to bowel gas and skin folds limiting visualization.

### Model performance: Associated angles

In two (33.3%) of the studies, the authors calculated angles and measurements not limited to limb length [17, 36]. Using their CNN, Larson et al. [17] also calculated the mechanical axis angle and pelvic tilt. The mechanical axis angle was calculated after connecting the automatically annotated key points at the femoral head apex, intercondylar notch, tibial spine and centre of the ankle mortise. Pelvic tilt was calculated after drawing horizontal annotations starting from the key point annotated at the ischium. With this approach, the authors reported MAEs of 0.42° for the mechanical axis angle and 0.67° for the pelvic tilt. Excellent reliability (ICC = 0.98) was reported for the mechanical axis angle prediction, whereas the reliability for pelvic tilt was considered good (ICC = 0.86).

Using LAMA, Simon et al. [36] were able to report the following measurements in addition to the length: hip–knee–ankle angle, anatomical‐mechanical‐angle, joint‐line‐convergence‐angle, mechanical‐lateral‐distal‐femoral‐angle, mechanical‐lateral‐distal‐tibia‐angle, mechanical‐lateral proximal‐femoral‐angle, mechanical‐medial proximal‐tibia‐angle and mechanical‐axis‐deviation. By localizing anatomic features of the femur, tibia and calibration ball automatically, the system was able to report these additional measurements with good to excellent reliability (ICC ≥ 0.87).

### Model performance: Efficiency

Four (66.6%) of the studies reported results regarding the efficiency/reading time of their models [11, 18, 39, 41]. In their study, Kim et al. [11] report that the radiologists spent, on average, 7 h and 12 min annotating LLD measurements. In comparison, their model spent 7 min and 26 s (approximately 62 times shorter. They also proposed a deep‐learning‐assisted approach, in which the radiologist manually reviews each machine‐predicted annotation and can accept or manually alter the resulting segmentations. With this approach, the radiologist only spent 74 min revising unsatisfactory cases. Of note, the authors did not specify the graphics processing unit (GPU)/processing capabilities used to annotate the radiographs.

Lee et al. [18] reported that a mean machine‐reading time of 8.68 ± 0.18 s per radiograph when using a single NVIDIA Titan‐XP GPU (NVIDIA). The authors did not provide a manual reading time for comparison.

Simon et al. [36] reported that LAMA (62 ± 5.3 s per radiograph) was three times faster than the manual measurements (192 ± 22.0 s per radiograph) in performing their array of measurements. They specify that LAMA was run on a laptop running Ubuntu Linux 18.04 LTS with a 4‐core Intel i7 (4600U 2.1 GHz) and 12 GB of RAM.

Tsai [39] reported an average run time of 0.26 s for each LLR. The author did not provide manual annotation times for comparison. The study was conducted using an Enkefalos 2.0 (Enkefalos Technologies LLP) cluster (28 nodes with 750 CPU cores, 13 GPUs and 3.5 TB memory) built on Intel architecture with a CentOS 7.6 Linux (CentOS Project) operating system.

The model developed by Zheng et al. [41] took approximately 1 s per radiograph, compared to 96 s per radiograph when calculated manually. Their CNN was trained and implemented using a GeForce GTX 1080Ti 11.0GB graphics processing unit (NVIDIA).

### Quality assessment

The average modified Methodological Index for Nonrandomized Studies score among all studies was 6.0 (0–7 scale) (Table 2), indicating excellent external validity of methodologic quality of the included studies on average.

**Table 2:** QUADAS report of studies included in methodological analysis.

	D2	D2	D3	D4	D5	D6	D7	Overall
Zheng et al. [41]	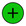	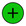	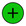	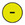	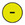	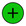	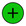	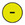
Lee et al. [18]	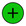	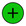	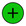	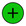	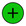	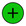	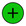	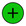
Simon et al. [36]	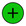	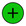	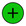	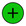	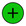	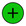	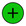	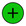
Tsai et al. [39]	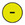	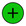	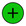	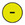	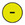	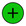	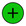	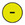
Kim et al. [11]	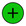	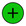	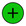	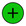	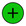	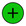	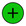	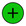
Larson et al. [17]	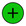	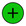	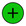	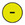	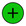	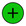	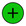	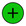
	D1: Patient selection (risk of bias)							Judgement
	D2: Index test (risk of bias)							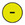 Unclear 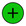 Low
	D3: Reference standard (risk of bias)							
	D4: Flow and timing (risk of bias)							
	D5: Patient selection (applicability)							
	D6: Index test (applicability)							
	D7: Reference standard (applicability)						

Abbreviation: QUADAS, quality assessment of diagnostic accuracy of studies.

## DISCUSSION

The present study aimed to review the current literature on the use of DL algorithms for the assessment of lower limb length and LLD using standardized AP LLRs.

The results of the present review suggest that DL algorithms can provide accurate and reliable measurements of lower limb length and LLD on LLRs. The reported MAE and ICC values indicate excellent agreement between the radiologist measurements and the DL model predictions, suggesting that DL algorithms could potentially be used to automate the measurement of lower limb length and LLD in clinical practice. Five of the studies reported femoral length, tibial length and leg length as separate measurements with associated performance metrics. Three of these studies (60%) reported MAE as a performance metric when comparing model performance to the ground truth labels, while the remaining two studies (40%) reported ICC values to indicate agreement between radiologist measurements and machine predictions. The reported MAE values ranged from 0.11 to 0.45 cm for femoral length, tibial length and leg length, while the reported ICC values for measurements of length ranged from 0.98 to 1.0, indicating excellent agreement between the radiologist measurements and the DL model predictions. In addition to quantifying individual bone length and full‐leg length, three (50%) of the studies reported measurements of LLD directly, with associated performance metrics. Tsai used the difference between the predicted and ground truth values for femur and tibia length to calculate LLD, reporting an MAE of 0.30–0.33 cm on their testing data set. Zheng et al. also reported an MAE of 0.45 for femur length, tibial length and full‐leg length on their testing data. Meanwhile, Liu et al. used the iliac crest height difference to quantify LLD, reporting a sensitivity of 0.90 and a specificity of 0.94 for their DL model.

The variability in DL architectures and training data sets used in the studies highlights the need for further research to optimize DL models for lower limb length and LLD assessment on LLRs. The methods used in the studies varied, with different DL architectures and training data sets employed. Larson et al. used a Faster‐RCNN—ResNet 101 model from the open‐source TensorFlow Object detection library, which has been successfully used in the past for automatic detection of knee osteoarthritis. Simon et al. used the LAMA software system for their analysis of 295 LLRs. Lee et al. described a cascade DL architecture composed of three stages that they used to assess LLD, using a single‐shot multibox detector to detect 12 ROIs, followed by 12 separate U‐net models to segment each ROI, and finally, an image‐processing algorithm to identify relevant landmarks in the pelvis, knee and ankle. Tsai used a CNN and k‐fold cross‐validation to evaluate model performance, while Zheng et al. used a CNN for coarse and fine segmentation and measured leg length by calculating the distance between the extracted corresponding pixels on the edges of the segmentation masks.

Finally, it will be important to validate the performance of DL algorithms on larger and more diverse data sets, as well as compare the performance of DL models with that of traditional measurement techniques in clinical practice. Further studies can also explore the potential of DL algorithms to identify specific anatomical landmarks and detect other lower limb abnormalities, which could further enhance the clinical utility of DL‐based lower limb length and LLD assessment.

## LIMITATIONS

First, there was no quantitative meta‐analysis performed as the data from each paper was not amenable which limits the ability to make statistical inferences about the overall performance of the DL models. Second, only a small number of studies (six) satisfied inclusion criteria which limits the generalizability of the findings. Third, there was heterogeneity in the DL models used and the inclusion/exclusion criteria across the studies, which makes it difficult to compare the results and draw firm conclusions about the optimal approach for lower limb length and LLD assessment using DL algorithms.

## CONCLUSIONS

DL algorithms show potential for accurate and reliable measurement of lower limb length and LLD on LLRs, with comparable performance to radiologists. Overall, DL algorithms have the potential to improve the accuracy, efficiency and consistency of lower limb length and LLD assessment in clinical practice.

## AUTHOR CONTRIBUTIONS

**Bradley A. Lezak**: Conceptualization; data curation; formal analysis; investigation; methodology; project administration; supervision; visualization; writing—original draft; writing—review and editing. **James A. Pruneski**: Conceptualization; data curation; formal analysis; investigation; methodology; project administration; supervision; visualization; writing—original draft; writing—review and editing. **Jacob F. Oeding**: Conceptualization; data curation; formal analysis; investigation; methodology; project administration; supervision; visualization; writing—original draft; writing—review and editing. **Kyle N. Kunze**: Supervision; visualization; writing—review and editing. **Riley J. Williams**: Supervision; visualization; writing—review and editing. **Michael J. Alaia**: Supervision; visualization; writing—review and editing. **Andrew D. Pearle**: Supervision; visualization; writing—review and editing. **Joshua S. Dines**: Supervision; visualization; writing—review and editing. **Kristian Samuelsson**: Supervision; visualization; writing—review and editing. **Ayoosh Pareek**: Supervision; visualization; writing—review and editing.

## CONFLICT OF INTEREST STATEMENT

The authors declare no conflict of interest.

## ETHICS STATEMENT

The authors have nothing to report.
